# Compression of the Left Brachiocephalic Vein by a Type II Right Aortic Arch: A Rare Vascular Anomaly With Unique Clinical Presentation

**DOI:** 10.7759/cureus.103572

**Published:** 2026-02-13

**Authors:** Ali Hamade, Mahmoud Awti, Kassem Haidar, Hasan Tarhini, Tony E Bechara

**Affiliations:** 1 Cardiology, Lebanese University Faculty of Medicine, Hadath, LBN; 2 Emergency Medicine, Lebanese University Faculty of Medicine, Hadath, LBN; 3 Cardiology and Echocardiography, Lebanese Hospital Geitawi, University Medical Center, Beirut, LBN; 4 Cardiology and Echocardiography, Central Military Hospital, Beirut, LBN

**Keywords:** aberrant left subclavian artery (alsa), congenital aortic arch anomaly, kommerell's diverticulum, left brachiocephalic vein compression, right-sided aorta

## Abstract

Right-sided aortic arch (RAA) is an uncommon anatomical variation of the thoracic vasculature, occurring in approximately 0.1% of adults. In nearly half of these instances, the left subclavian artery follows an abnormal course. The left subclavian artery typically emerges from a tapered dilation at its origin from the aorta, referred to as Kommerell’s diverticulum (KD). Based on available literature, only a few cases have been documented.

We describe a 62-year-old female patient who presented with a painless chest bump persisting for four weeks. She reported no symptoms such as cough, shortness of breath, fainting, or difficulty swallowing. Upon chest inspection, the middle of the chest exhibited a mild protrusion. Her vital signs were stable, and both pulmonary and cardiovascular examinations were normal. After an initial workup, a CT angiogram of the chest was performed and revealed the presence of a RAA compressing the left brachiocephalic vein (LBCV ) in addition to an aberrant left subclavian artery (ALSA).

In adults, right-sided aortic arch with an aberrant left subclavian artery originating from KD is a rare but typically silent anomaly. However, it can become clinically significant if complications arise, such as severe aneurysmal changes or compression of nearby mediastinal structures. Patients may then present with chest pain or dyspnea, coughing, and swallowing issues. In this case, the anomaly led to compression of the left brachiocephalic vein, resulting in its dilation and visible protrusion.

Although standardized treatment guidelines are lacking, it is important to educate patients about the nature and potential risks of this condition. For those without symptoms, regular monitoring may be appropriate, with surgical intervention considered if complications emerge.

## Introduction

Right-sided aortic arch (RAA) is an uncommon congenital configuration of the thoracic aorta, originally described by Fioratti and Aglietti in 1763, and identified in roughly 0.1% of adults [1]. About two out of five patients with this anomaly have an accompanying aberrant left subclavian artery (ALSA) [2]. In these instances, the altered vessel most often arises from a localized outpouching of the aortic wall, referred to as Kommerell’s diverticulum (KD) [3]. According to published reports, an estimated 50-80 cases combining RAA and ALSA originating from a KD have been documented [4].

## Case presentation

A 62-year-old female patient presented with a chest protrusion at the clinic. She was a smoker and had chronic hypertension treated with valsartan/hydrochlorothiazide 160 mg/12.5 mg, amlodipine 5 mg daily, and bisoprolol 5 mg. Her past surgical history included a spinal surgery for herniated disc disease localized in her lumbar vertebrae 10 years ago. At this visit, she mentioned that the protrusion had appeared about four weeks earlier. She denied any associated pain, discomfort, or dyspnea. The protrusion was not affected by any position. The vital signs were in normal range. Thoracic examination showed a central chest hump at the level of the second and third ribs toward the right of the sternum. No thrill nor bruit was noticed. No change in skin color was noticed. Pulmonary, cardiovascular, and laryngeal examinations were unremarkable. The complete blood count (CBC), electrocardiogram (EKG), and echocardiography showed no abnormality.

A chest X-ray performed revealed a widened superior mediastinum. The trachea was shifted toward the left. Both the lung parenchyma and the pulmonary vasculature appeared unremarkable. The aortic arch and descending thoracic aorta were visualized to the right of the vertebral column.

**Figure 1:**
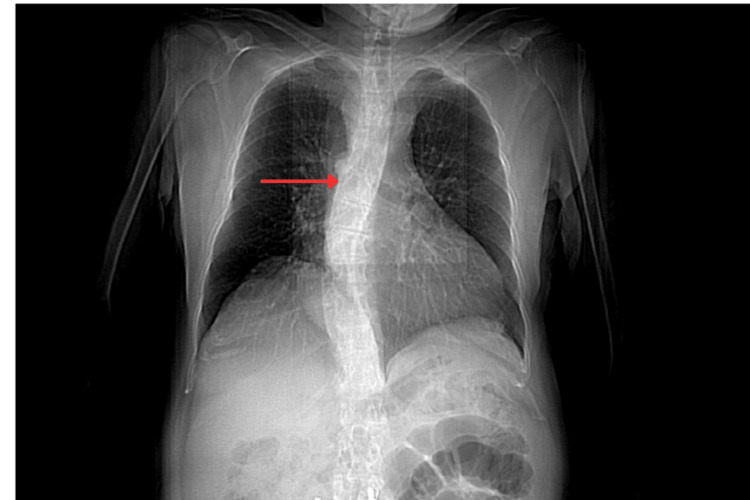
Coronal view of a CT chest showing the deviated descending aorta (red arrow)

**Figure 2:**
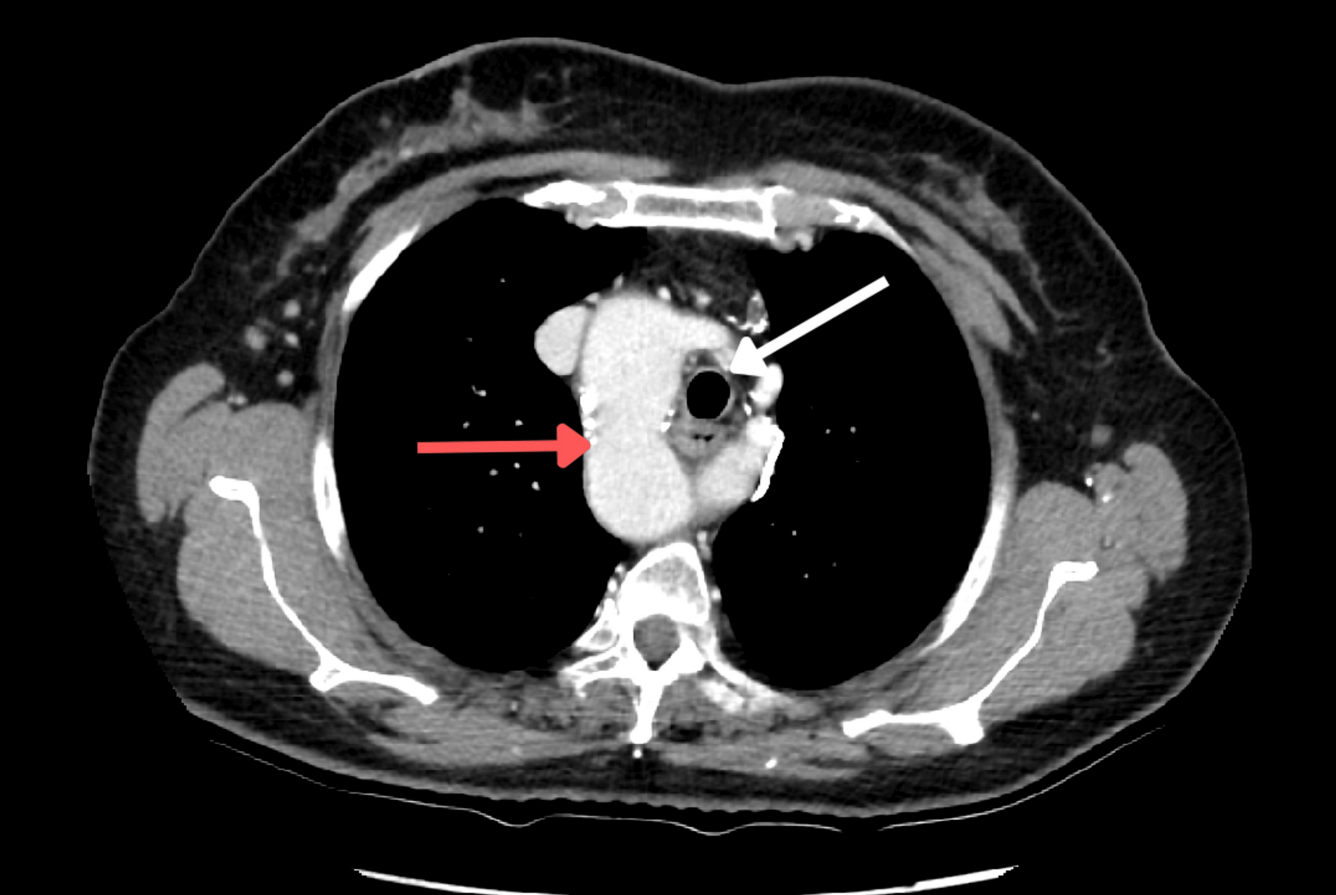
Axial view of a CT scan showing the right aortic arch (red arrow) to the right of the trachea (white arrow)

Based on the chest X-ray findings raising suspicion for RAA, further evaluation with contrast-enhanced chest CT was done. Coronal and axial reconstructions were utilized to better delineate the mediastinal vascular anatomy. The CT scan demonstrated that the ascending aorta arched to the right of the trachea (Figures 1, 2 ) and followed a deviated course toward the left. The aortic arch gave rise to the left common carotid artery, right common carotid artery, and right subclavian artery sequentially from proximal to distal.

**Figure 3:**
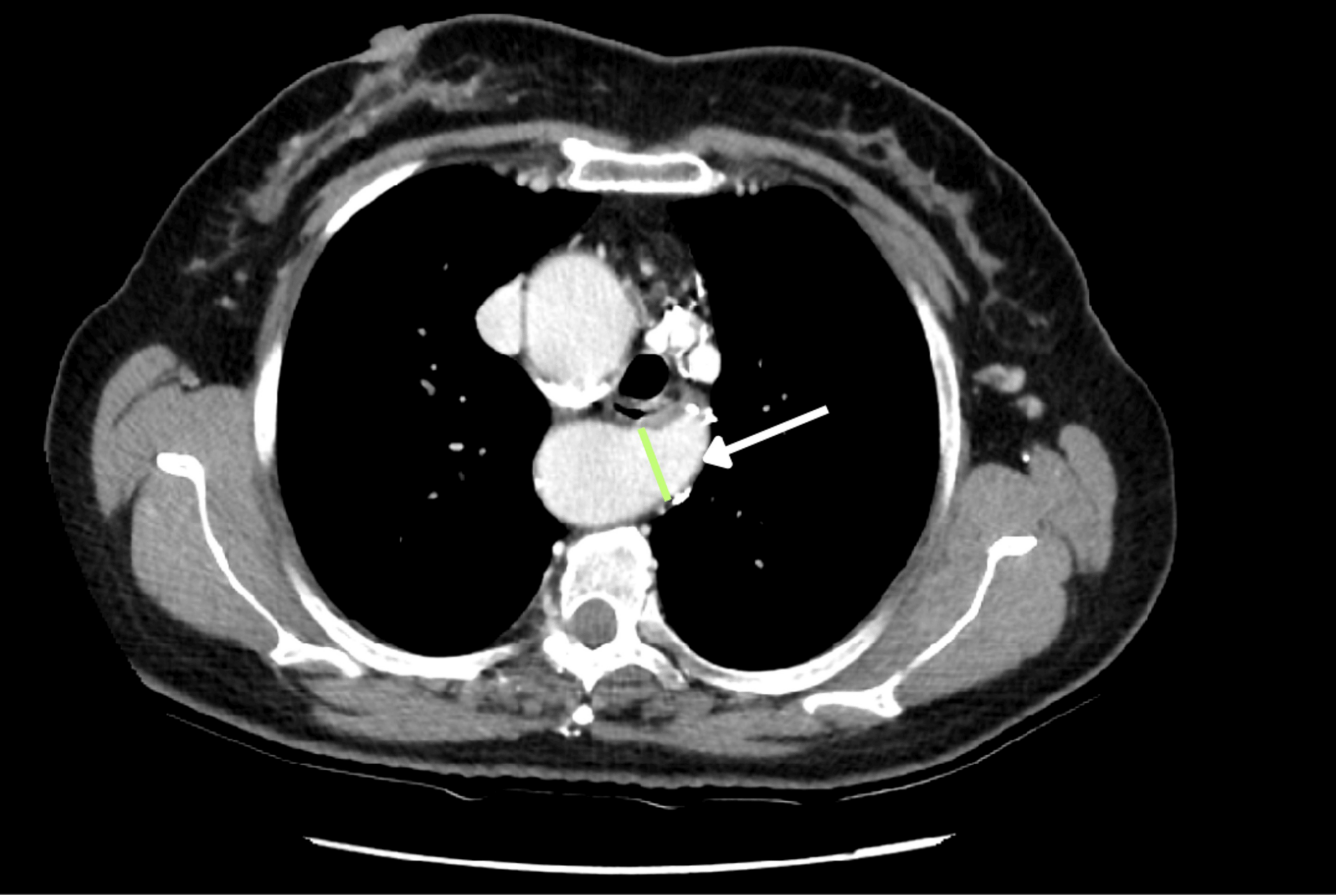
Axial view of the CT scan showing KD (green arrow) and the left aberrant subclavian artery (white arrow) KD: Kommerell’s diverticulum

The left subclavian artery was observed originating near the distal portion of the aortic arch, displaying a bulbous appearance at its origin. It coursed posterior to both the trachea and esophagus, crossing the midline toward the left and causing posterior indentation of these structures. This focal dilation at the artery’s origin (referred to as KD) measured 17 mm in its anteroposterior diameter (Figure 3).

**Figure 4:**
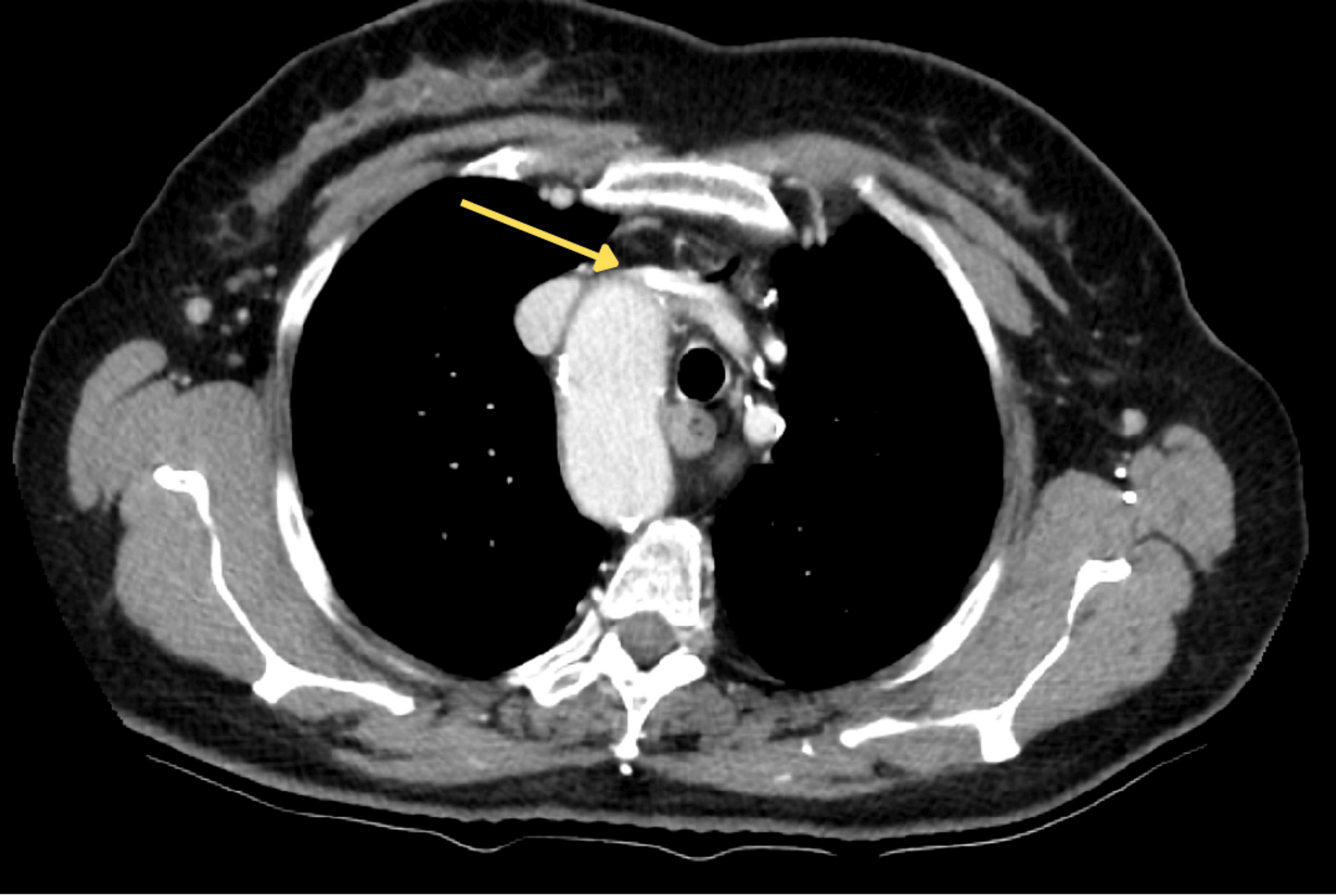
Axial view showing the compression of the left brachiocephalic vein (yellow arrow)

The thoracic aorta initially descended along the right side of the vertebral column before gradually shifting leftward as it approached the diaphragmatic hiatus. Adjacent to the diverticulum, the descending thoracic aorta reached a maximum diameter of 42.2 mm. Regarding the venous system, the left brachiocephalic vein (LBCV) was found to be compressed by the deviated aortic arch (Figure 4).

**Figure 5:**
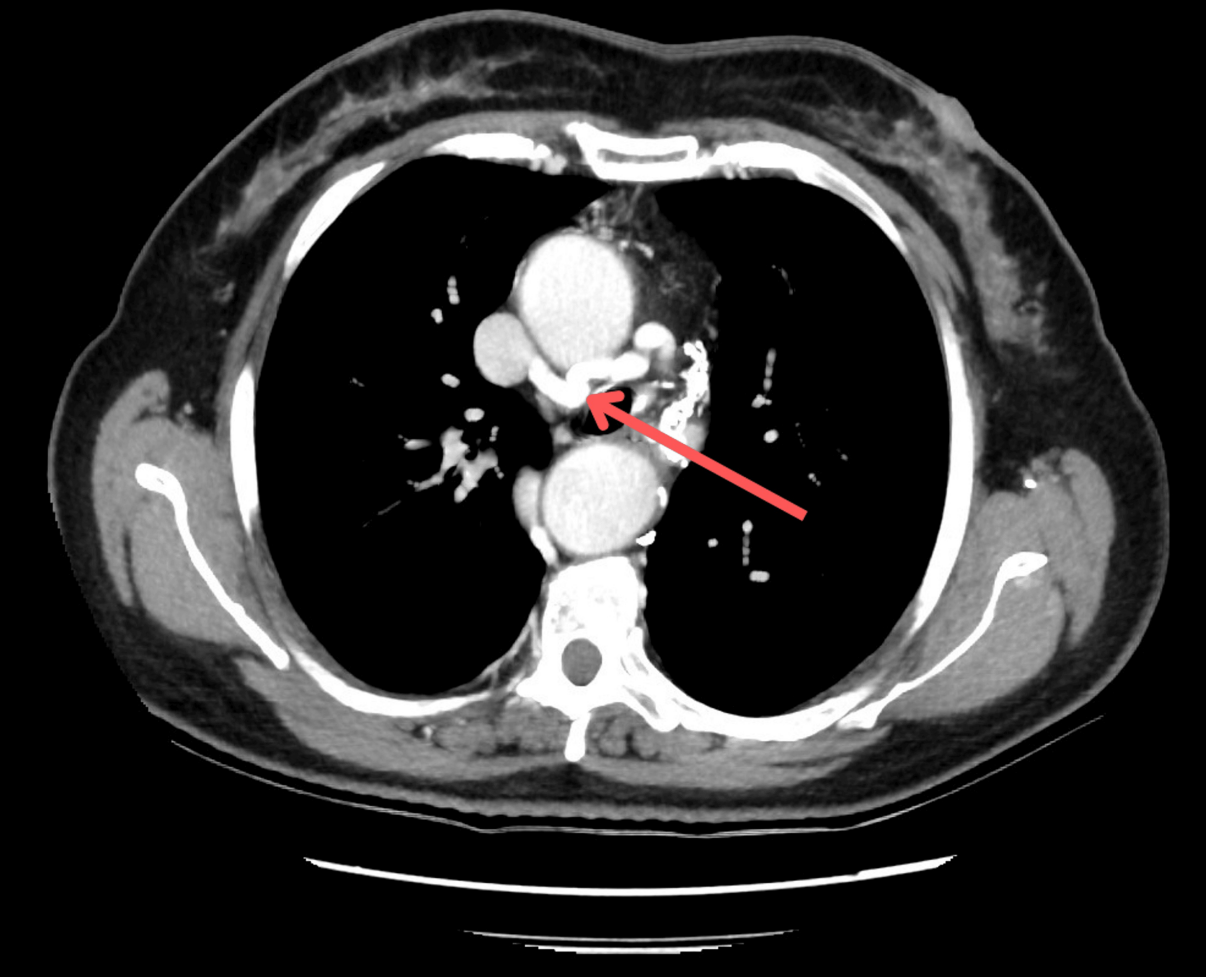
Axial view showing the collateral flow (red arrow) reaching the superior vena cava

Consequently, it drained into the superior vena cava via collaterals above the azygos vein (Figure 5). At the level of the middle mediastinum, a contralateral flow joined the superior vena cava above the azygos veins (Figure 5). The esophagus was minimally compressed.

Following a multidisciplinary team evaluation, the patient was diagnosed with a type II RAA anomaly, which was exerting compression on the LBCV. As the major mediastinal structures (including the trachea, esophagus, and pulmonary vessels) were unaffected, surgical intervention was deemed unnecessary. Further genetic testing was also considered unnecessary, as the patient’s phenotype did not suggest any chromosomal abnormalities.

## Discussion

In a normal anatomy, the aortic arch curves to the left of the trachea. During the development of the embryonic arch, it can abnormally deviate from the usual pattern and course to the right, forming an RAA. Most patients with this condition have no identifiable genetic alteration; however, this variant can be associated with chromosomal abnormalities such as 22q11 deletion (DiGeorge syndrome) and with congenital cardiac malformations, including tetralogy of Fallot, truncus arteriosus, and d-transposition of the great arteries [5].

In 1948, Edwards categorized RAA into three anatomical variants (type I, II, III ) based on the branching pattern of its vessels [6-8].

The most frequent form (type II) involves an ALSA originating from KD. Less commonly, the arch may exhibit mirror-image branching of the major arteries (type I) or complete isolation of the left subclavian artery collateralization (type III), both of which are often linked to cyanotic congenital heart conditions [9].

In the type II pattern, the first vessel to arise from the aortic arch is the left common carotid artery, which is followed in order by the right common carotid, the right subclavian, and lastly the ALSA. In this subtype, the descending thoracic aorta is usually positioned on the right side of the vertebral column or close to the midline. The ALSA often stems from a remnant of the left dorsal aorta, referred to as Kommerell's diverticulum. It was named for Burckhard F. Kommerell, a German radiologist who described it in 1936 after detecting an esophageal indentation caused by a pulsating mass on a barium swallow. This vascular arrangement develops when the proximal portion of the left fourth aortic arch regresses before the origin of the left subclavian artery [10].

RAA is often asymptomatic. In pediatric cases, clinical manifestations typically stem from coexisting congenital heart defects or from esophageal or tracheal compression caused by an ALSA. Among adults with type II RAA, symptoms occur in roughly 5% of cases and are generally attributed to pressure on adjacent structures, leading to issues such as difficulty swallowing, shortness of breath, or nonspecific chest discomfort [11].

Kommerell’s diverticulum can lead to serious complications, including diverticular rupture (4%), aortic dissection (11%), rupture of an aortic aneurysm, or rupture of an aberrant subclavian artery aneurysm. As these conditions are rare, standardized management guidelines are lacking. In general consensus, surgery is recommended when the diverticulum’s orifice diameter exceeds 30 mm and/or when the adjacent descending aorta measures more than 50 mm in diameter [12,13].

Returning to our case, as the left brachiocephalic vein drained into the superior vena cava through collateral pathways (thereby restoring venous return), surgical repair was not indicated. Neither anticoagulation nor antiplatelet therapy was recommended by our medical team, as no thrombosis was observed or suspected within the venous system. The exact mechanism and timing of this presentation at such an age remain unclear; however, the most likely explanation is age-related widening and structural remodeling of the aorta, which progressively compressed the LBCV.

The patient was advised to undergo regular follow-up and to report any alarming symptoms that had been explained prior to discharge.

## Conclusions

In conclusion, RAA represents a rare congenital anomaly, with an aberrant left subclavian artery being even less common. Although these vascular anomalies are uncommon, they may underlie a variety of clinical signs and symptoms. Therefore, heightened awareness of such conditions is essential for timely diagnosis and appropriate management. Notably, no documented cases of brachiocephalic vein compression secondary to RAA have been identified in the current literature. However, with the increasing accessibility and utilization of advanced imaging modalities, the incidental detection of RAA is becoming more frequent. Further research and comprehensive data are necessary to develop a standardized diagnostic and therapeutic algorithm for these anomalies.
